# Long-term Paleolithic diet is associated with lower resistant starch intake, different gut microbiota composition and increased serum TMAO concentrations

**DOI:** 10.1007/s00394-019-02036-y

**Published:** 2019-07-05

**Authors:** Angela Genoni, Claus T. Christophersen, Johnny Lo, Megan Coghlan, Mary C. Boyce, Anthony R. Bird, Philippa Lyons-Wall, Amanda Devine

**Affiliations:** 1 School of Medical and Health Sciences, Edith Cowan University, 270 Joondalup Drive, Joondalup, WA 6027 Australia; 2 School of Molecular and Life Sciences, Curtin University, Bentley, WA 6102 Australia; 3 School of Science, Edith Cowan University, Joondalup, WA 6027 Australia; 4 Forensic Biology, PathWest Laboratory Medicine, Nedlands, WA 6009 Australia; 5 CSIRO Health and Biosecurity, Gate 13, Kintore Avenue, Adelaide, SA 5000 Australia

**Keywords:** Paleolithic diet, Gut health, Resistant starch, TMAO, Whole grains

## Abstract

**Background:**

The Paleolithic diet is promoted worldwide for improved gut health. However, there is little evidence available to support these claims, with existing literature examining anthropometric and cardiometabolic outcomes.

**Objective:**

To determine the association between dietary intake, markers of colonic health, microbiota, and serum trimethylamine-*N*-oxide (TMAO), a gut-derived metabolite associated with cardiovascular disease.

**Design:**

In a cross-sectional design, long-term (*n* = 44, > 1 year) self-reported followers of a Paleolithic diet (PD) and controls (*n* = 47) consuming a diet typical of national recommendations were recruited. Diets were assessed via 3-day weighed diet records; 48-h stool for short chain fatty acids using GC/MS, microbial composition via 16S rRNA sequencing of the V4 region using Illumina MiSeq. TMAO was quantified using LC–MS/MS.

**Results:**

Participants were grouped according to PD adherence; namely excluding grains and dairy products. Strict Paleolithic (SP) (*n* = 22) and Pseudo-Paleolithic (PP) (*n* = 22) groups were formed. General linear modelling with age, gender, energy intake and body fat percentage as covariates assessed differences between groups. Intake of resistant starch was lower in both Paleolithic groups, compared to controls [2.62, 1.26 vs 4.48 g/day (*P* < 0.05)]; PERMANOVA analysis showed differences in microbiota composition (*P* < 0.05), with higher abundance of TMA-producer *Hungatella* in both Paleolithic groups (*P* < 0.001). TMAO was higher in SP compared to PP and control (*P* < 0.01), and inversely associated with whole grain intake (*r* = − 0.34, *P* < 0.01).

**Conclusions:**

Although the PD is promoted for improved gut health, results indicate long-term adherence is associated with different gut microbiota and increased TMAO. A variety of fiber components, including whole grain sources may be required to maintain gut and cardiovascular health.

**Clinical trial registrations:**

Australian and New Zealand Clinical Trial Registry (ANZCTRN12616001703493).

**Electronic supplementary material:**

The online version of this article (10.1007/s00394-019-02036-y) contains supplementary material, which is available to authorized users.

## Introduction

The Paleolithic diet is a dietary pattern based on the hypothesis that the human genome has not adapted to consume products of agriculture, and thus is based on consumption of meat, fish, eggs, nuts, fruits and vegetables; with no processed foods, grains or dairy products included [1]. The diet is promoted worldwide for improved gut health [2]. However, it excludes grains and dairy, food groups that form part of the evidence-based national Australian and international dietary guidelines [3, 4].

While total dietary fiber intake can be maintained on a Paleolithic diet through fruit and vegetable consumption [5], the exclusion of whole grains and legume products alters the fiber profile consumed, and in particular, results in reductions of resistant starch (RS) intake [6]. RS consistently improves markers of bowel health, such as increased SCFA levels [7–12], and long-term the effect of reduced intake has not been previously explored, nor the implications for microbial diversity, metabolites, and other markers of gastrointestinal health. While the Paleolithic diet can be classed as a low carbohydrate diet [5], other studies of low carbohydrate diets and the impact on markers for gastrointestinal health have been very low in total dietary fiber [13–16], thus limiting comparability to the current Paleolithic dietary patterns and the impact on markers of gut health.

The elimination of grains, dairy and legume protein sources, means PDs are rich in animal-based protein, which may increase serum trimethylamine-*N*-oxide (TMAO) concentrations [17]. TMAO has been associated with CVD and atherosclerotic plaque in both animal and human models [18–21], however, there is little evidence around how TMAO concentrations vary with total dietary patterns in healthy individuals. Given the established mechanism for the production of TMA within the colon [18], modulations of the gut microbiome through dietary intervention and changes in fiber intake have the potential to alter circulating TMAO concentrations. We have previously shown that a short-term, 4-week intervention using the PD, did not significantly impact TMAO, but lowered RS intake, in a small cohort of healthy Australian women [6]. However, longer-term studies of a PD have not examined the relationship between RS intake and TMAO concentrations [22–24]. Our short-term, randomised, controlled intervention study, comprised a small sample size and the energy restricted diet may have limited our ability to detect significant differences in TMAO concentrations [6]; furthermore, we did not concurrently examine fecal microbiota. Given the identified link between TMAO concentrations and CVD [18–21], and the limited literature regarding long-term health implications of the PD, it is important to determine if the Paleolithic dietary pattern alters the ability of the gut microbiota to produce TMA. Therefore, the current cross sectional study compared subjects with long-term (> 1 year) adherence to a Paleolithic diet to those following a standard Australian diet to examine the impact of each diet on gastrointestinal health and potential downstream effects on cardiovascular health.

## Methods

### Trial design

The study was designed as a cross-sectional comparator study, and registered on the Australian and New Zealand Clinical Trial Register (ANZCTRN12616001703493) and approved by the Edith Cowan University, Human Research Ethics Committee (13402).

### Participants

Recruitment for the study took place between August 2016 and June 2017 through online advertisements. Primary inclusion criteria for the Paleolithic diet group were adherence to the dietary pattern for > 1 year period and consumption of no more than 1 serve/day of grains and dairy products. For inclusion in the control group, participants needed to have made no changes to their diet in the previous year, and follow a relatively healthy diet which included grains, legumes and dairy or alternatives. Specific inclusion criteria for both groups were: men and women aged 18–70 years; willingness to complete a 3-day weighed diet records (3d WDR), provide blood, urine and stool samples; non-smoker, not participating in other studies and had BMI < 30 kg/m^2^. Subjects were excluded if they had taken antibiotics in the previous 6-month, had a past or present digestive disorder, surgery on the gastrointestinal tract, used anti-hypertensive or lipid or glucose-lowering medication, previous cardiovascular events or diagnosed CVD. Participants were screened via email or phone confirming exclusion/inclusion criteria were met and provided written informed consent prior to study commencement. Participants completed a diet history interview, followed by a 3d WDR, including 2 week days and 1 weekend day. Samples collected were a 24-h urine and fasted (overnight) blood sample. Portable freezers (Waeco-CF-40,Dometic, Australia) were supplied to collect all stool samples over a 48-h period. Physical activity was assessed by the International Physical Activity Questionnaire [25].

### Validity of dietary intake data

Dietary data provided by the 3d WDR were validated by urine nitrogen analysed using a 1 in 50 dilution of urine sample on a Shimadzu Total Carbon and Nitrogen Analyser, TOC-Vcsh/TMN-1 (Shimadzu, Japan). Total nitrogen intake was determined by dividing protein intake by 6.25 [26], with an acceptable intake to excretion ratio set at 80% ± 24% [27]. Those with an intake to excretion ratio outside of this range were deemed to be protein intake under or over reporters. Potential energy under reporters were identified utilising the Goldberg cut point [28]. Those who were identified as under reporting both protein and energy were defined as unreliable dietary reporters. Confirmation that the 3d WDR was representative of usual dietary intake was achieved by statistical analysis of the energy and protein intake of both methods.

### Paleolithic scoring protocol

Due to individual differences in interpretation of the Paleolithic dietary pattern noted during data-entry of the 3d WDR, a post hoc scoring protocol was developed to rank adherence to the Paleolithic diet principles, namely the exclusion of grain and dairy products. Those who consumed < 1 serve per day of grains and dairy, in-line with the inclusion criteria, were allocated to the Strict Paleolithic (SP), while those who consumed > 1 serve per day of grains and/or dairy were allocated to the Pseudo-Paleolithic group (PP).

### Outcomes assessment

#### Dietary intake

The diet history and 3d WDR data were entered into FoodWorks v8.0 [29], by the same assessor, a registered nutritionist with advanced competencies in dietary analysis. All food records were checked for completeness. Minimum and maximum RS content of each food item, utilised in the 91 food items generated from the 3d WDR, were determined using methods described elsewhere [6].

### Anthropometric measures

Subjects were fasted for 2-h prior to the clinic appointment and reported dressed in tightly fitting gym clothes. Blood pressure measurement was conducted, in duplicate, on the right arm with an Omron IA1B Automated Blood Pressure Device (Omron Health Care Ltd, Japan) at heart level, 1 min apart. The mean of the two systolic and diastolic measures respectively were recorded as per the protocol described by the American Heart Foundation [30]. Standing height to the nearest 0.1 cm was recorded using a SECA 763 digital stadiometer (SECA Ltd, USA). Waist circumference was measured to the nearest 0.1 cm at the narrowest part of the waist by a Lufkin steel tape measure, following standardised ISAK techniques [31]. The BodPod body composition chamber (Cosmed, USA) calculated both weight and body fat percentage (to the nearest 0.001 kg and 0.01%, respectively). As per the manufacturer’s protocol, hair was covered with a tightly fitted cap, with all jewellery and footwear removed.

### Stool form and biochemistry

Upon arrival at the university, the Bristol stool number (as reported by the participant at collection time [32]) was recorded. Each individual sample was weighed and the total number of samples provided over the 48-h period allowed the calculation of stool frequency (bowel motions/day).

Individual stool samples were stored at − 80 °C until sample processing, where they were defrosted at 4 °C overnight. Samples from the same participant were combined and homogenised manually on ice for at least 1 min prior to weighing and aliquoting for each individual assay. SCFA analysis was undertaken using 1–1.5 g aliquots of stool using methods described by Bajka et al. [33], with the addition of a freeze–thaw distillation prior to GC analysis.

Moisture content was calculated by freeze-drying, in duplicate, 40 g of pre-aliquoted stool sample for 7 days using a Christ LD-alpha freeze drier (Martin Christ Ltd., Germany). Moisture content, expressed as a percentage, was calculated from the mean of the two individual moisture measurements.

### Blood biochemistry

Participants reported to pathology for a blood test on the morning after completion of the 3WDR, after an overnight fast. Lipids were determined using standard enzymatic techniques (Abbott Architect c16000 assay) by Pathwest, a National Association of Testing Laboratories (NATA) accredited laboratory. Serum samples, stored at − 80 °C, were analysed for TMAO at the School of Science Analytical facility, Edith Cowan University, using the method described by Le et al. [34].

### Microbiota analysis

The microbial analysis were conducted at the WA Human Microbiome Collaboration Centre, Curtin University, utilising the QIAmp PowerFecal DNA Kit for DNA extraction and Illumina MiSeq platform for sequencing. Full methods are available in the supplementary information.

### Statistical methods

A priori power calculations were determined using G-Power software [35] and were based on reductions to our primary outcome variable, fecal butyrate excretion. Available literature suggested a medium to large Cohen’s effect size (*d* = 0.595) could be expected [13], providing sample size requirement of *n* = 72 at 80% power and *α* = 0.05, however, given fiber intake can be maintained on a Paleolithic diet, a more conservative estimate was determined using a medium effect size (*d* = 0.5). The actual sample requirement was therefore deemed to be between a total of *n* = 72 and *n* = 102, with *n* = 36 and *n* = 51 per group, respectively.

Data were analysed using SPSS v24.0 [36]. Non-normally distributed data were log_10_ transformed prior to analysis and back-transformed to allow calculation of the estimated marginal means and corresponding 95% confidence intervals. General linear modelling was used to compare the stratified Paleolithic vs control groups. Post hoc Bonferroni corrections were applied to *P* values from the three groups analysis. Amongst the entire cohort, exploration of associations between dietary intake, blood and stool biochemistry were conducted using linear regression. All models included age, gender, energy intake and body fat percentage as covariates, with additional covariates used where appropriate. Significance for the study was set at *P* < 0.05.

Microbiota analysis was conducted using Primer 7 (Quest Research, NZ) with permutation multivariate analysis of variance (PERMANOVA) [37]. At the phylum and genus level, relative abundance data were square or fourth root transformed, prior to the calculation of a Bray–Curtis similarity matrix. Principal coordinates analysis (PCO) was used to examine possible differences or separations among the groups visually at the 2-D level. This was followed by PERMANOVA to formally assess differences between groups. Dissimilarity percentage (SIMPER) analysis was used to determine the contribution of individual phyla and genera driving the average dissimilarities between groups based on the Bray–Curtis similarity matrix. Distance-based linear modelling (DistLM) was utilised to describe the patterns in the microbiota using the dietary intake variables. Measures of diversity were calculated using the Shannon and Simpson indices. The Bacteriodetes:Firmicutes ratio was calculated and exported to SPSS v24.0 (IBM Corporation, USA) [36] for analysis.

All analyses were conducted with and without the inclusion of participants identified as unreliable dietary reporters. Where the inclusion of these participants did not influence statistical significance of our findings, the reported results include all participants.

## Results

### Participants

**Fig. 1:**
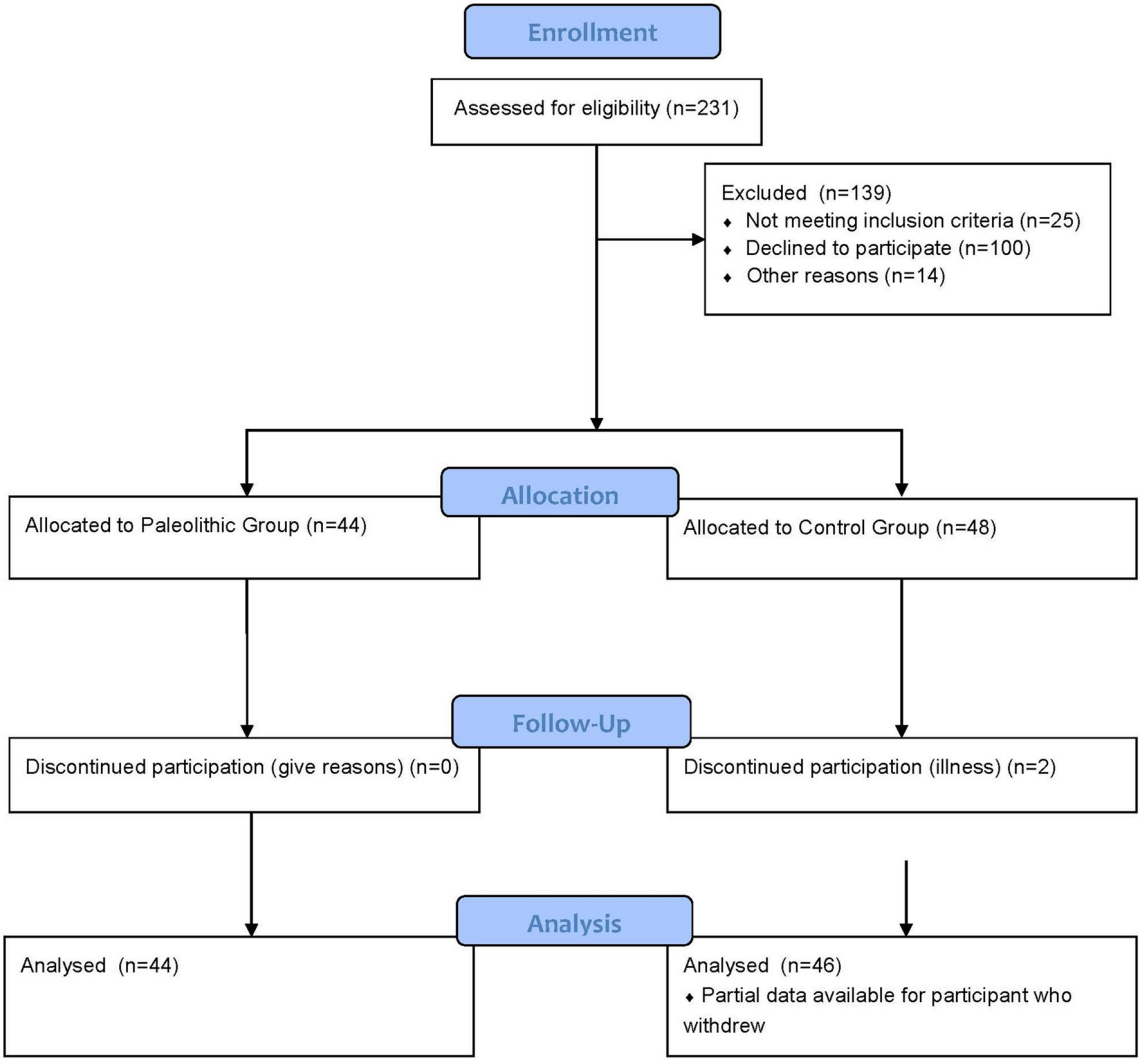
Flow diagram of the study. Of the 231 enquiries received, 92 participants were accepted into the study. Two participants in the control group withdrew due to illness, with partial data available for one subject. 44 Paleolithic and 46 controls completed the study data collection

As shown in Fig. 1, 90 subjects completed the study with partial data available for an additional control subject. Participant characteristics are presented in Table 1. No significant differences were found between groups for age, height, energy intake or physical activity. The average length of time Paleolithic participants had followed the diet was 2.38 ± 1.08 years. In terms of being representative of long-term dietary intake, bivariate correlations of energy and protein with the diet history showed good agreement (*R* = 0.596 and 0.763 respectively, *P* < 0.01) and a one sample *t* test showed the energy and protein intake determined from the 3d WDR and the diet history were not significantly different (*P* = 0.623 and 0.059 respectively). A Bland–Altman plot [38] confirmed acceptable agreement between urinary nitrogen and calculated nitrogen intake from protein intake (data not shown); and a one-sample *t* test showed no difference between these variables (*P* = 0.337). Five participants were identified as under-reporting both energy and protein intake, two from the SP group and three from the control group. The scoring protocol was used to rank participants according to adherence to the diet (Table 1). Body weight was significantly greater in the Pseudo-Paleolithic (PP) group, when compared to the controls, but there were no other differences between groups identified.

**Table 1:** Participant characteristics by dietary group

	Control Mean (95% CI)	Strict Paleolithic (SP) Mean (95% CI)	Pseudo-Paleolithic (PP) Mean (95% CI)
*N*	47	22	22
Age (years)^a^	38.96 (35.02, 42.90)	41.49 (35.46, 47.53)	45.33 (39.69, 50.96)
Height (cm)	169.6 (167.9, 171.4)	169.8 (167.1, 172.5)	172.8 (170.3, 175.3)
Weight (kg)	69.19 (65.58, 72.79)	70.23 (64.74, 75.72)	79.08 (73.89, 84.27)**
BMI (kg/m^2^)	23.94 (22.78, 25.10)	24.33 (22.56, 26.10)	26.39 (24.72, 28.07)
Systolic blood pressure (mmHg)	120 (114, 125)	116 (109, 124)	114 (107, 125)
Diastolic blood pressure (mmHg)	79 (75, 83)	75 (70, 81)	77 (72, 82)
Waist circumference (cm)	81.3 (78.1, 84.5)	83.0 (78.2, 87.8)	86.1 (81.7, 90.6)
Body fat (%)	22.68 (20.21, 25.14)	22.38 (18.63, 26.13)	25.96 (22.41, 29.50)
Energy intake (kJ)	9384 (8800, 9968)	9171 (8282, 10059)	9812 (8972, 10652)
Physical activity (PAL)^b^	1.82 (1.79, 1.86)	1.87 (1.81, 1.93)	1.83 (1.77, 1.86)

**P < 0.01 different from control group. Means calculated using general linear modelling with the addition of age and gender as covariates

^a^Unadjusted mean

^b^PAL Score derived from PAL ranking (from IPAQ Scoring Protocol) [40] and converted to PAL score [41]

### Dietary intake

Total dietary fiber (TDF) intake in the SP group was not different from that of the control (Table 2), however the PP group had fiber intake below the recommended Australian adequate intake (AI) of 25 g/day. In line with the significantly lower TDF intake in the PP group, both soluble and insoluble fibre intake was also lower, when compared to the control group. RS intake was significantly lower in the SP and PP groups at both minimum and maximum estimated intakes (Table 2). Despite the low carbohydrate intake, vegetable intake in the SP group met the Australian recommendations of 5 serves per day [3], with a mean intake significantly higher than that of the control group. Across the whole cohort, total fiber intake was inversely associated with body weight [*t*(86) = − 4.22, partial *r* = − 0.414, *P* < 0.001), body fat percentage [*t*(86) = − 2.282, partial *r* = − 0.239, *P* = 0.025] and waist circumference [*t*(85) = − 3.397, partial *r* = − 0.346, *P* = 0.001]. Protein intake as percentage of energy and absolute intake in grams per day, was significantly higher in the SP group, when compared to the control and PP (Table 2). Fat intake was significantly higher in both Paleolithic groups, when compared to the control (Table 2). Saturated fat intake in all dietary groups exceeded the recommended intake of 10% of energy [3], with intakes in both of the Paleolithic groups more than double the recommended Australian and international levels [3, 39].

**Table 2:** Energy, macronutrient and food group intake by dietary group

Dietary variable	Control Mean (95% CI)^a,b^ (*n* = 47)	Strict Paleolithic Mean (95% CI)^b^ (*n* = 22)	Effect size (SP vs control)	Pseudo-Paleolithic Mean (95% CI)^b^ (*n* = 22)	Effect size (PP vs control)	Overall dietary effect *P* value^c^
Energy intake (kJ)	9187 (8599, 9774)	8955 (8076, 9834)	− 0.13	9820 (9005, 10635)	0.33	0.285
Protein (g)	92.16 (83.69, 100.63)	118.07 (105.69, 130.44)**	1.02	102.7 (90.62, 114.77)	0.39	**0.001**
Protein (% of energy)	18.49 (16.87, 20.12)	23.63 (21.25, 26.00)**	1.02	20.13 (17.81, 22.45)	0.39	**0.001**
Carbohydrate (g)	202.62 (187.84, 217.41)	99.19 (77.60, 120.79)**	− 2.33	81.40 (60.34, 102.47)**	− 2.59	**< 0.001**
Carbohydrate (% of energy)	38.03 (35.49, 40.57)	16.75 (13.03, 20.46)**	− 2.79	16.41 (12.79, 20.03)**	− 2.69	**< 0.001**
Sugars (g)	75.58 (66.05, 85.11)	51.77 (37.85, 65.68)**	− 0.83	44.34 (30.76, 57.91)**	− 1.04	**< 0.001**
Starch (g)	124.56 (112.87, 136.26)	45.86 (28.78, 62.95)**	− 2.24	34.74 (18.07, 51.4)**	− 2.43	**< 0.001**
Total fat (g)	82.53 (76.33, 88.72)	117.91 (108.86, 126.96)**	1.90	133.16 (124.34, 141.99)**	2.58	**< 0.001**
Fat (% of energy)	36.45 (33.9, 39)	52.95 (49.22, 56.67)**	2.15	56.96 (53.33, 60.6)**	2.54	**< 0.001**
Saturated fat (g)	27.34 (22.77, 31.9)	45.51 (38.84, 52.18)**	1.33	52.47 (45.97, 58.98)**	1.74	**< 0.001**
Saturated fat (% of energy)	12.02 (10.05, 13.99)	20.59 (17.71, 23.47)**	1.45	22.09 (19.28, 24.9)**	1.61	**< 0.001**
Polyunsaturated fat (g)	15.17 (13.21, 17.14)	16.86 (13.98, 19.73)	0.29	17.47 (14.67, 20.28)	0.37	0.295
Monounsaturated fat (g)	32.96 (29.63, 36.28)	45.01 (40.16, 49.86)**	1.21	52.28 (47.55, 57.02)**	1.84	**< 0.001**
Cholesterol (mg)	324.19 (225.29, 423.08)	708.28 (563.84, 852.73)**	1.29	685.00 (544.10, 825.90)**	1.15	**< 0.001**
Total dietary fiber (g)	29.66 (26.36, 32.96)	27.41 (22.59, 32.23)	− 0.23	20.79 (16.09, 25.49)*	− 0.85	**0.006**
Soluble dietary fiber (g)	11.99 (10.45, 13.53)	10.78 (8.54, 13.03)	− 0.26	7.62 (5.43, 9.81)**	− 0.90	**0.004**
Insoluble dietary fiber (g)	17.07 (15.11, 19.03)	16.17 (13.3, 19.04)	− 0.15	12.87 (10.07, 15.66)*	− 0.68	**0.036**
Resistant starch minimum (g)	4.48 (3.89, 5.06)	2.62 (1.77, 3.47)**	− 1.03	1.26 (0.43, 2.09)**	− 1.75	**< 0.001**
Resistant starch maximum (g)	14.16 (12.24, 16.07)	6.13 (3.34, 8.93)**	− 1.35	2.95 (0.22, 5.68)**	− 1.85	**< 0.001**
Alcohol (g)	7.07 (3.73, 10.4)	8.31 (3.44, 13.19)	0.12	10.34 (5.59, 15.09)	0.31	0.488
Vegetables serves/day (fresh equivalent 150–350 kJ)	3.83 (2.99, 4.66)	6.68 (5.51, 7.84)**	1.12	4.31 (3.17, 5.44)	0.16	**< 0.001**
Dark green vegetable serves/day (fresh equivalent 100 kJ)	0.39 (0.17, 0.61)	1.28 (0.97, 1.59)**	1.34	0.70 (0.41, 1.00)	0.45	**< 0.001**
Nuts and seeds (30 g protein equivalent)	0.82 (0.48, 1.15)	1.07 (0.59, 1.56)	0.26	1.32 (0.84, 1.79)	0.48	0.167
Red meat serves/day (20 g protein equivalent)	0.44 (0.21, 0.66)	0.80 (0.47, 1.13)	0.52	0.77 (0.45, 1.09)	0.46	0.066
Eggs serves/day (13 g protein equivalent)	0.20 (0.09, 0.30)	1.05 (0.72, 1.38)**	0.87	0.89 (0.57, 1.21)*	0.63	**< 0.001**
Total grains serves/day (14–16 g starch equivalent)	6.14 (5.29, 7.04)	0.19 (0.03, 0.47)**	− 3.80	0.63 (0.30, 1.08)**	− 3.07	**< 0.001**
Whole grain serves/day (14 g starch equivalent)	2.91 (2.28, 3.61)	0.09 (0.00, 0.34)**	− 2.32	0.16 (0.01, 0.46)**	− 2.09	**< 0.001**
Total dairy serves/day (300 mg calcium equivalent)	1.60 (1.25, 2.0)	0.17 (0.04, 0.37)*	− 1.26	0.87 (0.54, 1.27)*	− 0.73	**< 0.001**

*P* values for stratified Paleolithic groups were adjusted using post hoc Bonferroni correction [43]

Significant *P* values are in bold

^a^General linear modelling used to determine estimated marginal means and the difference between control and the overall Paleolithic groups after adjustment for age, gender, energy intake and body fat percentage.**P ≤ 0.01 different from control group, *P ≤ 0.05 different to control group

^b^General linear modelling used to determine estimated marginal means and the difference between control, strict Paleolithic and Pseudo-Paleolithic groups after adjustment for age, gender, energy intake and body fat percentage.**P ≤ 0.01 different from control group, *P ≤ 0.05 different to control group. *P* < 0.01 different from Strict Paleolithic group, *P* < 0.05 different from the SP group

^c^Significance of the overall general linear model for the three group analysis. Effect sizes reported using Cohen’s *d*, with a value of 0.2 representing a small effect, 0.5 a medium effect and 0.8 a large effect

### Blood biochemistry

Total serum cholesterol was higher in the PP, when compared to the control group (Table 3). High-density lipoprotein (HDL) cholesterol was significantly higher in the SP and PP groups, when compared to the control group and led to non-significant differences among the groups in the total cholesterol:HDL cholesterol ratio (Table 3). Total serum cholesterol concentrations were inversely associated with carbohydrate intake, both expressed as g/day [*t*(77) = − 2.47, partial *r* = − 0.271, *P* = 0.016] and as a percentage of energy [*t*(77) = − 2.54, partial *r* = − 0.280, *P* = 0.013]. Further exploration showed significant negative association with the minimum [*t*(77) = − 3.329, partial *r* = − 0.355, *P* = 0.001], and maximum RS intakes [*t*(77) = − 2.922, partial *r* = − 0.316, *P* = 0.005] and whole grain intake [*t*(77) = − 2.032, *r* = − 0.226, *P* = 0.046].

**Table 3:** Biochemistry results by dietary group

Biomarker	Control Mean (95% CI)^a,b^ (*n* = 46)	Strict Paleolithic Mean (95% CI)^b^ (*n* = 22)	Effect size (strict Paleolithic vs control)	Pseudo-Paleolithic Mean (95% CI)^b^ (*n* = 22)	Effect size (Pseudo-Paleolithic vs control)	Overall dietary effect *P* value^c^
*Blood biochemistry*						
Total Cholesterol (mmol/L)	4.88 (4.51, 5.26)	5.02 (4.48, 5.56)	0.13	5.60 (5.10, 6.11)*	0.65	**0.050**
LDL Cholesterol^d^ (mmol/L)	3.00 (2.70, 3.30)	3.10 (2.66, 3.53)	0.12	3.49 (3.09, 3.90)	0.55	0.113
HDL Cholesterol (mmol/L)	1.43 (1.29, 1.57)	1.69 (1.49, 1.89)**	0.64	1.79 (1.61, 1.98)*	0.88	**0.003**
Total Cholesterol/HDL Ratio	3.49 (3.21, 3.77)	3.14 (2.73, 3.55)	− 0.41	3.25 (2.87, 3.64)	− 0.28	0.122
Triglycerides (mmol/L)	0.94 (0.88, 1.0)	0.86 (0.79, 0.94)	− 0.49	0.84 (0.77, 0.91)	− 0.60	**0.049**
Serum TMAO (µM)^e^	3.93 (2.79, 5.55)	9.53 (5.86, 15.49)**	0.86	5.47 (3.42, 8.75)	0.32	**0.008**
*Stool markers*						
Stool weight (g/day wet weight)	194.57 (161.7, 227.43)	248.81 (200.55, 297.06)	0.55	233.04 (186.03, 280.05)	0.37	0.099
Frequency (motions per day)	1.37 (1.19, 1.54)	1.60 (1.35, 1.85)	0.46	1.91 (1.67, 2.16)**	1.03	**0.001**
Bristol number [31]	4.12 (3.75, 4.48)	4.3 (3.76, 4.84)	0.18	4.54 (4.02, 5.07)	0.38	0.338
Moisture (%)	73.96 (72.04, 75.89)	74.69 (71.86, 77.52)	0.12	74.5 (71.74, 77.26)	0.09	0.880
pH^e^	7.19 (7.05, 7.36)	7.19 (6.97, 7.41)	− 0.33	7.18 (6.97, 7.4)	− 0.06	0.974
Residual fecal fat (%)^e^	1.09 (0.69, 1.5)	1.16 (0.57, 1.75)	0.07	1.70 (1.13, 2.27)	0.49	0.165
Acetate excretion (mmol/day)^e^	7.80 (6.18, 9.86)	10.52 (7.46, 14.86)	0.41	9.06 (6.49, 12.68)	0.19	0.288
Propionate excretion (mmol/day)^e^	2.36 (1.87, 2.98)	2.76 (1.96, 3.89)	0.80	2.82 (2.02, 3.94)	0.70	0.546
Isobutyrate excretion (mmol/day)^e^	0.27 (0.23, 0.32)	0.34 (0.26, 0.44)	0.42	0.34 (0.27, 0.44)	0.41	0.152
Butyrate excretion (mmol/day)^e^	2.21 (1.72, 2.84)	2.86 (1.98, 4.13)	0.33	2.85 (1.99, 4.07)	0.32	0.315
Isovalerate excretion (mmol/day)^e^	0.39 (0.33, 0.46)	0.45 (0.36, 0.58)	0.29	0.48 (0.38, 0.61)	0.39	0.255
Valerate excretion (mmol/day)^e^	0.30 (0.25, 0.36)	0.37 (0.29, 0.49)	0.40	0.38 (0.29, 0.49)	0.41	0.136
Caproate excretion (µmol/day)^e^	0.05 (0.04, 0.08)	0.06 (0.03, 0.11)	0.05	0.08 (0.04, 0.15)	0.28	0.618
Total SCFA excretion (mmol/day)^e^	13.74 (11.02, 17.14)	17.91 (12.94, 24.83)	0.38	16.41 (11.94, 22.54)	0.25	0.299

Significant *P* values are in bold

^a^General linear modelling used to determine estimated marginal means and the difference between control and the overall Paleolithic groups after adjustment for age, gender, energy intake and body fat percentage.**P ≤ 0.01 different from control group, *P ≤ 0.05 different from control group

^b^General linear modelling used to determine estimated marginal means and the difference between control, strict Paleolithic and Pseudo Paleolithic groups after adjustment for age, gender, energy intake and body fat percentage.**P ≤ 0.01 different from control group, *P ≤ 0.05 different from control group

^c^Significance of the overall general linear model for the three group analysis. Effect sizes reported using Cohen’s *d*, with a value of 0.2 representing a small effect, 0.5 a medium effect and 0.8 a large effect

^d^LDL Cholesterol reported is calculated LDL. *P* values for stratified Paleolithic groups adjusted using post hoc Bonferroni correction [43]

^e^Log transformation conducted prior to analysis

TMAO was significantly higher in the SP group, when compared to the control group (Table 3). TMAO concentrations were associated with servings of red meat [*t*(67) = 3.372, partial *r* = 0.357, *P* = 0.001], and inversely associated with total grain [*t*(83) = − 3.143, partial *r* = − 0.335, *P* = 0.002] and whole grain intake [*t*(83) = − 2.973, partial *r* = − 0.319, *P* = 0.004], respectively.

### Stool biochemistry

There was no difference in daily stool weight or form but stool frequency was greater in the PP group when compared to the control group (Table 3). Regression analysis across the whole cohort showed stool frequency was significantly associated with nut and seed consumption after adjustment for energy intake [*t*(87) = 2.14, partial *r* = 0.223, *P* = 0.036], with each serve of nuts increasing motions/day by 0.12. There were no significant differences in SCFA excretion between groups, however, total SCFA excretion was associated with fiber intake [*t*(84) = 2.331, partial *r* = 0.247, *P* = 0.022], but not with carbohydrate (expressed as a percentage of energy and g/day), grains, whole grains, or RS intake. Total dietary fibre predicted butyrate excretion [*t*(84) = 2.232, partial *r* = 0.237, *P* = 0.028] but no relationships with wholegrains, grains and resistant starch were noted. Vegetable intake significantly predicted total SCFA excretion [*t*(84) = 2.27, partial *r* = 0.240, *P* = 0.026] and further analysis showed the association was due intakes of the dark green vegetable group [*t*(84) = 2.047, partial *r* = 0.218, *P* = 0.044], with similar associations seen for acetate excretion; vegetables were associated with acetate excretion [*t*(84) = 2.69, partial *r* = 0.281, *P* = 0.009], and subgroup analysis showed the association was due to the dark green vegetables [*t*(5,84) = 2.52, partial *r* = 0.265, *P* = 0.014].

### Microbiota profiles

The sequence data yielded 929 denoised operational taxonomic units (OTU) with an average 38,899 reads per sample (ranging from 2647 to 107,597 reads). Two participants from the control group were inadvertently tagged with identical forward and reverse barcodes and were excluded from the analysis. OTU comprising less than 0.01% of the total reads were eliminated (514 OTU), leaving 415 available for analysis. Five sequence controls comprised total reads between 3409 and 11,560. Five OTU not related to the gut microbiota, were present primarily in the blank samples, totalling 11,210 reads and were removed from the analysis. One OTU (432 reads) comprised human mitochondrial DNA and were also excluded from the analysis. The final dataset included 410 total OTU’s, which was converted to relative abundance before importing to PRIMER 7 [37] for analysis.

The microbial composition was analysed both at the phylum and at the genus level to examine major and more specific shifts in the gut microbiota between the three dietary groups. At the phylum level, there was no difference between groups for measures of diversity (Shannon and Simpson indices) or species richness between the three groups (Table S1). However, PERMANOVA analysis at the phylum level showed significant differences in microbiota composition between the control and PP groups (*P* = 0.048) and the SP and PP groups (*P* = 0.001). No difference was observed between the SP and control groups. SIMPER analysis indicated that group differences were driven by the relative abundances of Bacteriodetes, Firmicutes and Verrucomicrobia phyla. The relationship between Bacteriodetes and Firmicutes has been linked with increased body fat [40, 41], therefore, the overall Bacteriodetes:Firmicutes ratio was calculated, and between-group differences were evaluated using general linear modelling, with the addition of age, gender, energy intake and body fat percentage as covariates in the model. No differences were observed between the three groups. Although, in the entire cohort body fat percentage (partial *R* = − 0.33, *P* = 0.032) and energy intake (partial *R* = − 0.23, *P* = 0.033) were significant predictors of the ratio in a linear regression model.

At the genus level, there were no significant differences between the three groups, in measures of Shannon or Simpson diversities, and species richness. PERMANOVA analysis with covariates age, gender, energy intake and body fat percentage, showed there were significant differences between the SP and control (*P* = 0.004) and PP and control (*P* = 0.04), with no significant difference between the two Paleolithic groups.

**Fig. 2:**
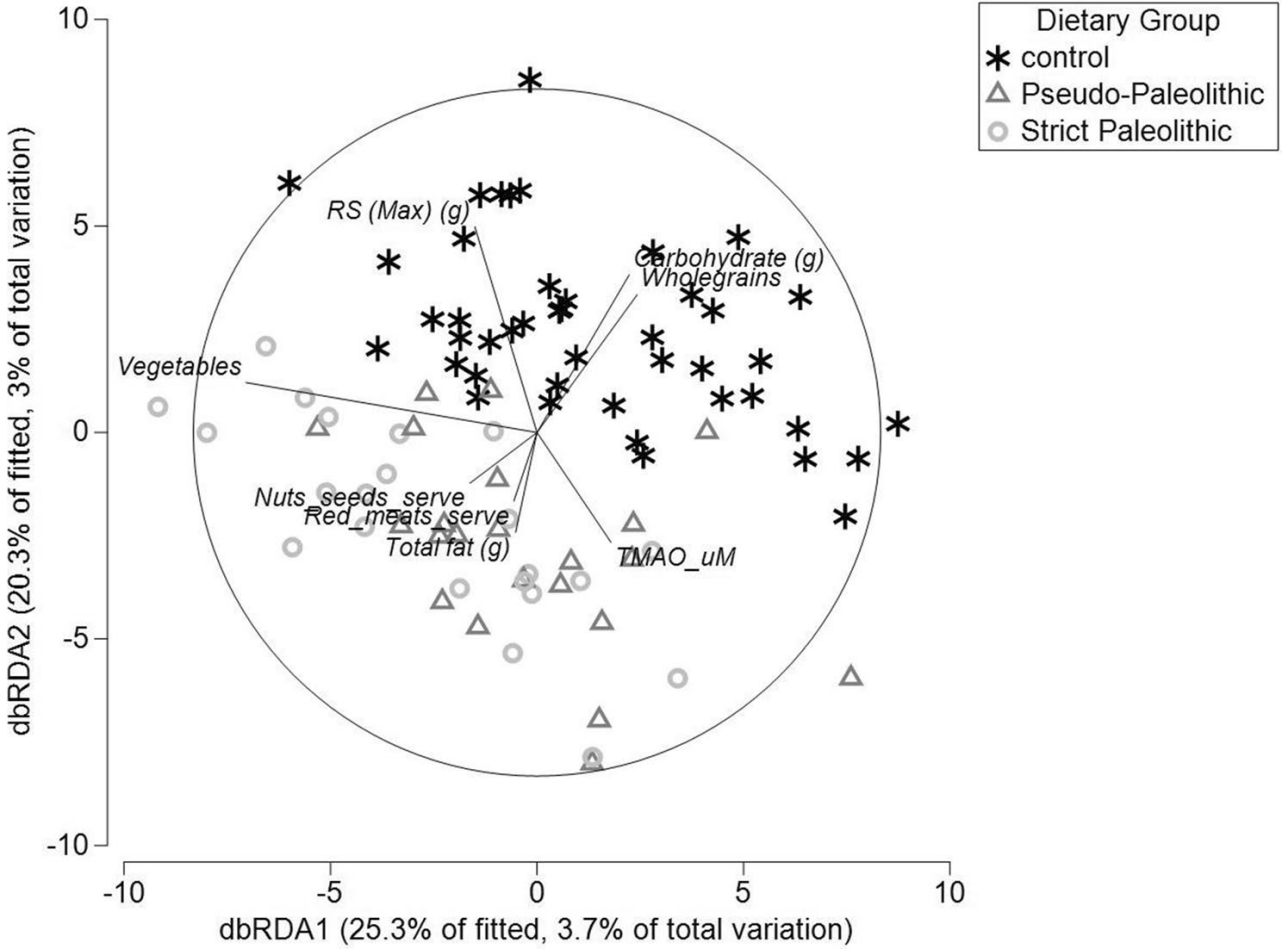
Distance-based redundancy plot, showing the effect of dietary intake, overlaid with serum TMAO, and microbiota composition. The added dietary variable vectors show the correlation between the dietary factor and the microbiota composition. The length of the line is indicative of the strength of the association. Whole grains (*P* < 0.01), carbohydrate (*P* < 0.01) and RS (*P* < 0.01) are associated with a shift in composition towards the top of the plot, consistent with microbiota composition of the control group. However, dietary fat (*P* = 0.04) was associated with a shift in the opposite direction, with vegetables (*P* < 0.01) falling in between the two groups. Consistent with the serum TMAO increase in the SP group, TMAO is closely aligned with microbiota composition, total fat and red meat consumption (*P* trend = 0.09)

DistLM showed a significant influence of vegetable intake (*P* < 0.01), in addition to significant influences of whole grains (*P* < 0.01), RS maximum estimation (*P* < 0.01), total carbohydrate (*P* < 0.01) and total fat (*P* = 0.04) on the microbial composition (Fig. 2).

**Fig. 3:**
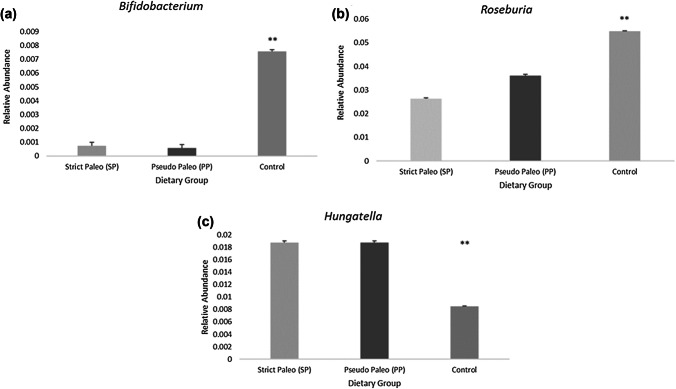
**a**–**c** Significant contributors to genera differences between groups. Bar graphs show relative abundances by dietary group. **P < 0.01 difference between control and PP/SP groups

Having established significant differences at the genera level, SIMPER analysis was used to determine the species which contributed to the differences identified between groups. The average dissimilarity between SP and control groups was 27.25%, SP and PP 26.27% and PP and control 27.13%. Abundant genera driving dissimilarities between groups were *Bacteriodes*, *Faecalibacterium*, *Blatia*, *Ruminoclostridium*, *Alistipes*, *Roseburia*, *Ruminococcus*, *Lachnospiracea Incertae sedis*, *Ruminoclostridium*, *Anaerostipes*, *Gemmiger*, *Irregularibacter*, *Akkermansia*. After adjustment for the study covariates, age, gender, energy intake and body fat percentage, three of these genera were identified as being significantly different between groups, *Bifidobacteria*, *Roseburia*, and *Hungatella* (Fig. 3a–c).

Relative abundance of *Hungatella* was significantly and inversely associated with grain consumption in a linear regression model after adjustment for age, gender, body fat and stool frequency (partial *r* = − 0.298, *P* = 0.006). Relative abundance of *Roseburia* was significantly related to total grain (partial *r* = 0.315, *P* = 0.002), whole grain (partial *r* = 0.298, *P* = 0.004) and carbohydrate consumption (partial *r* = 0.297, *P* = 0.004), but not with dietary fiber, or RS minimum or maximum estimates. Similar to *Roseburia*, *Bifidobacteria* abundance was related to total grain consumption (partial *r* = 0.266, *P* = 0.008) and the percentage of energy derived from carbohydrate (partial *r* = 0.221, *P* = 0.028), but not with RSi, whole grains or total dietary fiber. In a linear regression model, relative abundances of *Roseburia* and *Bifidobacteria* were significantly and inversely correlated with the abundance of *Hungatella* [*t*(87) = − 2.1, partial *r* = − 0.2, *P* = 0.03]. In line with the TMAO findings, relative abundance of *Hungatella* was also significantly associated with servings of protein foods per day [*t*(87) = − 2.84, partial *r* = 0.27, *P* = 0.006].

## Discussion

This study evaluated the gastrointestinal implications of low carbohydrate, high fat, Paleolithic style diets through comparison with a cohort of healthy Australians in a cross-sectional study design. Consumption of a long-term Paleolithic diet was associated with markedly higher serum TMAO concentrations, but only in those who adhered to the diet strictly. Romano et al. [42], identified six species of bacteria associated with choline consumption and production of TMA, of which, one was identified in our cohort, *Clostridium hathewayi*, originating from the genera *Hungatella.* We did not identify the other species reported by Romano et al. [42], although our methods of short amplicon sequencing are not typically used to identify individual species, but rather provide robust data at the genus level. It is therefore possible the other species were present, but not identified at the species level in our cohort. Nonetheless, the relative abundance of the *Hungatella* genus was significantly higher in both the SP and PP groups. Our results show that serum TMAO concentrations and *Hungatella* abundance were inversely associated with total and whole grain consumption, indicating these food groups may downregulate the ability of *Hungatella* to dominate or interfere with the production of TMA. Notably, TMAO concentrations in the PP group were not statistically different from the controls, despite the increased *Hungatella* abundance and small to medium effect size noted. The stratification of the Paleolithic group into two groups may have reduced our ability to detect significance of this outcome variable. Furthermore, the lower overall fiber and higher fat content of the PP diet may have influenced the fermentative capacity of the microbiota to produce TMA, given high fat diets may attenuate the fermentation response [43].

Bergeron et al. [44] detected changes in TMAO concentrations after a 2-week dietary intervention with 52 subjects and found low carbohydrate diets, high in RS, were associated with increased plasma TMAO. Conversely, in the current study TMAO was not associated with RS, but inversely associated with grain intake. This may indicate that other components of the grain carbohydrate and fiber are responsible for modulating abundances of *Hungatella*. Bergeron et al. [44] did not report food group intake for the intervention diets, which limits comparability with our study. In support of the findings presented here is the identification of the genera associated with producing TMA in the fecal microbiota [42], in addition to the statistical association found with red meat intake, a known TMA precursor food [45].

While there were no observed differences in measures of fecal microbiota diversity (α-diversity), a significant group difference (β-diversity) at both the phylum and genus levels were reported. An inverse association was found between the Bacteriodetes:Firmicutes ratio and body fat, supporting previous research showing a reduced ratio was associated with obesity [40, 41, 46]. At the genus level, different community structures were associated with intakes of vegetables, dietary fat, RS, whole grain and dietary fiber. The direction of the shift in microbiota composition was similar for vegetable intake, whole grains, RS and dietary fiber and is likely to be beneficial, given the large body of evidence associated with health benefits from consumption of these food groups [3, 47–54]. Conversely the direction of the shift in microbiota composition associated with fat consumption was in the opposite direction and suggests a more deleterious profile. Reductions to core bacteria including *Roseburia*, as seen in the current study, have been associated with inflammatory bowel diseases [55]. In animal models, high-fat diets have been shown to drive obesity independently of the composition of the microbiota [56]. There were reductions in the Paleolithic dietary groups to genera *Roseburia* and *Bifidobacterium*, known to metabolise carbohydrate and produce butyrate. Moreover, low abundances of known beneficial genera such as *Bifidobacterium* in the Paleolithic groups support the findings of Brinkworth et al. [13], who reported low abundances of this genera after an 8 week low-carbohydrate diet, comprising 58% fat [13]. A decrease in relative abundance of *Bifidobacterium* has been previously associated with irritable bowel syndrome, and obesity [57], however, these disorders have also been associated with lower fecal excretion of acetate and butyrate, which was not found in the current cohort. Given that total fat intake was associated with microbiota composition at both the phylum and genus level, and positively correlated with body weight, the differences observed in microbiota composition are unlikely to be beneficial.

Supporting our previous findings from a short-term intervention using the Paleolithic diet [6], the elimination of the grains food group on a Paleolithic diet resulted in a significantly lower intake of RS than the control group, despite no significant differences found in total soluble or insoluble fiber intakes. Intakes of RS in the control group were slightly higher than previous estimates of Australian intakes of 3.4–9.4 g/day [58] and may be due to higher consumption of whole grains than the average Australian intake of 1.5 serves/day [59]. Both groups consumed less than the proposed 20 g RS/day required for bowel health [60] and may be an area for focus in future interventions.

Despite the differences in RS intake, we did not observe any differences between groups in SCFA excretion. While this was an unexpected finding, the high intake of saturated fat in the Paleolithic groups may have influenced the results, given Fava et al. [61], showed fecal SCFA concentrations were higher following a 4-week diet comprising 17% saturated fat in 88 participants at risk of metabolic syndrome. It is not yet understood whether saturated fat leads to changes in fermentation patterns or alterations to colonic uptake of SCFA. Differences in microbiota composition attributed to the intervention diets used by Fava et al. [61] were assessed using fluorescence in situ hybridisation (FISH), which limits comparability with the current study. In addition, the lack of difference we observed for SCFA excretion may have been due to the differences in the sites of fermentation for grains as opposed to vegetables, with the latter likely to be fermented in the distal colon due to the difference in fiber structure, although there is little literature in this area. Even though fecal acetate concentration has been inversely associated with acetate absorption [62] fecal SCFA may not provide a sensitive enough marker to estimate differences in total fermentation along the colon. [63].

Previous short-term interventions using the Paleolithic diet have found increases to HDL cholesterol in diabetics [64], but not in healthy populations [5, 65–67], however, the latter also reported reductions to total and LDL cholesterol, in addition to significant weight loss, which may have influenced HDL concentrations. Further, a 2-year intervention showed no significant change in total or HDL cholesterol, despite significant reductions in triglycerides over the intervention period [23]. The current data showed small, but significantly greater HDL concentrations in the Paleolithic groups, which were associated with the reduction in carbohydrate intake, and increase in saturated fat consumption. The findings reported here support previous work showing that saturated fat consumption increases concentrations of calculated HDL cholesterol [68]. The current study also shows a significant relationship between saturated fat consumption and total cholesterol levels. A systematic review and meta-analysis showed that for every 1 mmol/L increase in total cholesterol, the relative risk of CVD was 1.20 for women (95% CI 1.16, 1.24) and 1.24 in men (1.20, 1.28) [69]. Therefore, while there may be conflicting evidence surrounding the specific effect of saturated fat consumption on CVD risk, the positive association noted in the current data between saturated fat and HDL concentrations must be interpreted with caution, as saturated fat intake was also associated with total cholesterol concentrations in the Paleolithic groups and may result in increased CVD risk over a longer-term period. Furthermore, we found a positive association between saturated fat intake, body weight, and BMI, which are known to increase CVD risk [70]. Taken together with the greater observed serum TMAO concentrations, it cannot be concluded that the Paleolithic diet is associated with improved gut health and a reduction in risk of CVD as promoted [71, 72].

## Conclusion

The cross-sectional data collected suggests that long-term adherence to the Paleolithic diet may not be beneficial for gut health, due to the association with lower relative abundances of known beneficial bacterial genera, and the increased relative abundance of TMA producing genera *Hungatella*. Our findings highlight that further research is required to understand the role of vegetables and saturated fat and how they influence colonic uptake of SCFA and subsequent excretion. The rationale to exclude whole grains is not supported by data presented here; being inversely associated with body weight and TMAO concentrations. Despite the maintenance of SCFA excretion, and stool frequency and form, the differences noted in microbiota composition associated with the high fat and low carbohydrate intake may not be beneficial for long-term health.

## Electronic supplementary material

Below is the link to the electronic supplementary material.

Supplementary material 1 (DOCX 33 kb)

## Abbreviations

TMAO: Trimethylamine-*N*-oxide
SP: Strict Paleolithic group
PP: Psuedo-Paleolithic group
PD: Paleolithic diet
RS: Resistant starch
