# Supplementary material for: Long-term Paleolithic diet is associated with lower resistant starch intake, different gut microbiota composition and increased serum TMAO concentrations
Source: Eur J Nutr. 2019 Jul 5;59(5):1845–58. doi: 10.1007/s00394-019-02036-y (PMC7351840; doi:10.1007/s00394-019-02036-y)
Supplement: Supplementary file 1 — Supplementary material 1 (DOCX 33 kb) [file 394_2019_2036_MOESM1_ESM.docx]

Supplementary Information

## DNA Extraction and Sequencing Method

The QIAamp PowerFecal DNA Kit (QIAGEN, Netherlands) was utilised for the DNA extraction following the manufacturer instructions, using 0.2 g of well-homogenised sample, and two 30-second intervals of beat-beating using a Bertin mini-lyser (Bertin Instruments, France) for mechanical lysis. Five QC samples (no stool) were extracted at the same time and carried all the way through to sequencing to control for introduced contamination in the extraction and sequencing workflow. DNA concentrations were determined spectrophotometrically, using the QIAxpert (QIAGEN, Netherlands). PCR inhibitors were assessed by a three point dilution of each sample prior to quantitative polymerase chain reaction (qPCR) using same cycling conditions and primers as described below. For the amplicon PCR, 2 µL of the highest non-inhibited DNA dilution and 23 µL master mix were combined (14.45 µL ultrapure water, 2.5 µL reaction buffer (Applied Biosystems [ABI], USA), 2 µL magnesium chloride (ABI), 1 µL bovine serum albumin (Fisher Biotec, Aus), one µL 10 µM forward primer, one µL 10 µM reverse primer (Integrated DNA Technologies [IDT] Aus), 0.6 µL SYBR (Invitrogen, USA), 0.25 µL dNTP’s (25 mM dATP, 25 mM dTTP, 25 mM dCTP, 25 mM dGTP; Astral Scientific, Aus) and 0.2 µL Ampli*Taq* Gold (ABI). Primers amplifying the V4 region of the 16S rRNA gene (515) were used. The forward primer (GTGCCAGCMGCCGCGGTAA), was combined with a seven base forward multiplex identifier (MID) barcode, and the reverse primer (GGACTACHVGGGTWTCTAAT) with an eight base MID barcode. The combination of barcodes was unique for each sample. PCR amplification was performed using a StepOnePlus Real-Time PCR System (Life Technologies, Thermo Scientific, USA). Samples were mini-pooled in equal proportion based on ΔRn florescent values prior to purification using QIAquick PCR purification kit (QIAGEN, Netherlands). All minipools were then combined based on amplicon concentration prior to PCR-free ligation based on the method by Kozarewa and Turner, (2011). The ligation process involved an end-repair reaction for 30 minutes at 20°C in a thermal cycler (DNA Engine, BioRad) using End-repair enzyme mix, End-repair reaction buffer (New England Biolabs, [NEBNext], USA) and ultra-pure PCR water to a final reaction volume of 100 μL. The end-repaired mixture was purified using the QIAquick PCR purification protocol (Qiagen) and eluted in 32 μL of EB buffer. The amplicon pool then underwent an A-tailing reaction in a 50 μL volume with 5 U/μL Klenow exo (3’-5’ exo minus), 10X Klenow buffer, and dATP (NEBNext) for 30 minutes at 37°C in a thermal cycler (DNA Engine). After A-tailing, the amplicon pool was purified and concentrated down again (QIAquick PCR purification, Qiagen) and eluted in 30 μL of EB buffer. The pool was then quantified on the QIAxpert (Qiagen) to determine the volume of adapter required for a 3:1 adapter to sample ratio. The ligation reaction was then carried out in a 50 μL total volume with 28 μL of amplicon pool, Quick T4 DNA Ligase (NEBNext), 5X Quick Ligation Reaction Buffer (NEBNext), phosphorylated adapters (IDT), and ultra-pure PCR water for 15 minutes at 20°C in a thermal cycler (DNA Engine). The ligated products were purified again (QIAquick PCR purification, Qiagen), eluted in 30 μL of EB buffer, and size selected to remove any residual adapter dimer using a Pippin Prep 2% agarose with ethidium bromide and external marker B cassette (Sage Science, USA). The size selected amplicon pool was purified a final time (QIAquick PCR purification, Qiagen) to remove ethidium bromide attained during size selection, and eluted in 40 μL of EB buffer. The ligation library amplicon pool was serially diluted in EB buffer, and quantified by qPCR against a standard 200 bp synthetic oligonucleotide (IDT) of known molarity. The qPCR result was used to approximate the amount of pooled ligation library required for successful clustering on the MiSeq Flowcell (Illumina, USA). The MiSeq sequencing set-up was carried out as per the manufacturers protocol. The amplicon library was bidirectionally sequenced using a 500-cycle V2 reagent kit and a V2 Standard flowcell (Illumina).

The amplicon sequence data was processed using GHAP, an in-house amplicon clustering and classification pipeline built around tools from USearch [73] and RDP[74], combined with locally-written tools for demultiplexing and generating Operational Taxonomic Unit (OTU) tables. The amplicon reads were demultiplexed, and the read pairs are then merged and de-replicated. The merged reads were then trimmed and clustered at 97% similarity to generate OTUs. Representative sequences from each OTU were then classified both by finding their closest match in a set of reference 16S sequences and by using the RDP Naïve Bayesian Classifier. The OTU tables were summarised overall taxonomic levels, combining the counts for identified taxa across all OTUs. The pipeline classified all the merged reads using the RDP Classifier to provide confidence in the clustering and OTU formation steps.

## Supplementary Tables.

Table S1. Measures of diversity and richness by dietary group

| **Genus Composition Measure** | **Control group**  **Mean (95% CI)ᵟ**  **(n=44)** | **Strict Paleolithic groupᵟ**  **(n=22)** | **Effect Size (SP vs control)** | **Pseudo-Paleolithic groupᵟ**  **(n=22)** | **Effect Size (PP vs control)** | **P-Value^ϯ^** |
| --- | --- | --- | --- | --- | --- | --- |
| Shannon diversity index^π^ | 4.35 (4.29, 4.41) | 4.31 (4.26, 4.35) | -0.26 | 4.34 (4.28, 4.4) | -0.07 | 0.457 |
| Simpson diversity index | 1.04 (1.04, 1.04) | 1.04 (1.04, 1.04) | 0.00 | 1.04 (1.04, 1.04) | 0.00 | 0.302 |
| Species richness | 30.02 (28.53, 31.52) | 29.68 (28.63, 30.72) | -0.11 | 30.2 (28.75, 31.65) | 0.05 | 0.798 |

*ᵟGeneral Linear Modelling used to determine estimated marginal means and the difference between Control, Strict Paleolithic and Pseudo Paleolithic groups after adjustment for age, gender, energy intake and body fat percentage.**P≤0.01 Different from Control Group, *P≤0.05 Different from Control Group..**P≤0.01 Different from Control Group, *P≤0.05 Different from Control Group. ^ƚ^Significance of the overall general linear model for the three group analysis. Effect sizes reported using Cohen’s d, with a value of 0.2 representing a small effect, 0.5 a medium effect and 0.8 a large effect. ^π^log transformation conducted before analysis. P-Values for stratified Paleolithic groups adjusted using post-hoc Bonferroni correction.[75]*
